# Evaluation of the Effects of Bromelain and Papain Enzymes on Shear Bond Strength of Composite Resin to Enamel

**DOI:** 10.1155/2021/3233639

**Published:** 2021-07-12

**Authors:** Farahnaz Sharafeddin, Mohammad Hossein Yazdanpanah, Zahra Jowkar

**Affiliations:** ^1^Department of Operative Dentistry and Biomaterials Research Center, School of Dentistry, Shiraz University of Medical Sciences, Shiraz, Iran; ^2^Oral and Dental Disease Research Center, Department of Operative Dentistry, School of Dentistry, Shiraz University of Medical Sciences, Shiraz, Iran

## Abstract

**Aim:**

This study aimed to evaluate the effects of 6% bromelain and 10% papain enzymes on shear bond strength (SBS) of composite resin to enamel compared to conventional 37% phosphoric acid etching.

**Materials and Methods:**

50 human maxillary premolar teeth were randomly divided into 5 groups (G1–G5/*n* = 10). In G1 and G2, after etching enamel with 37% phosphoric acid for 15 seconds and washing the surface, 10% papain and 6% bromelain enzymes were used, respectively. In G3 and G4, 6% bromelain or 10% papain enzymes were applied on enamel. In G5, the enamel surface was etched with 37% phosphoric acid for 15 seconds. A two-step etch-and-rinse adhesive system (Adper Single Bond 2) was applied. A nanohybrid composite (Z350) was placed using Teflon molds. All the samples were then subjected to the SBS test using a universal testing machine. Data analysis was performed using a one-way ANOVA test followed by the Tukey test. *p* values less than 0.05 were considered significant.

**Results:**

Comparison of the mean SBS between G1, G2, and G5 shows no significant differences (*p* > 0.05); however, they had higher mean SBS compared with G3 and G4 (*p* < 0.0001).

**Conclusions:**

The shear bond strength of composite to enamel was not affected significantly using either 6% bromelain or 10% papain enzymes after 37% phosphoric acid application. Moreover, 6% bromelain and 10% papain enzymes were not as effective as 37% phosphoric acid alone.

## 1. Introduction

The mechanical properties of restorative materials and their long-term clinical performance are very important because they are continuously exposed to conditions that may affect their bond strength; these materials should have the ability to withstand high mechanical forces during mastication; therefore, the adequate strong bonding of the restorative materials to the tooth structure is the goal of adhesive dentistry [1]. Composite resins are considered the material of choice in restorative dentistry [2, 3]. The acid etching with phosphoric acid can change the enamel surface, rendering it more receptive to adhesion. The bond strengths of adhesive restorations to enamel augment when the enamel surface is deproteinized and the organic substances are eliminated before the acid etching process [4–6]. Papain, commonly known as the papaya fruit, is derived from the latex of *Carica papaya*, which belongs to the Caricaceae family. Papain shows an extensive proteolytic activity and can break down the organic substances and chemical removal of caries without damaging intact collagen fibrils [5, 7, 8]. Bromelain is yet another proteolytic enzyme derived from the stem, leaves, and fruit parts of the pineapple (*Ananas comosus*) and other species of the Bromeliaceae family [4, 9]. Recently, several studies have evaluated the effects of deproteinization with papain and bromelain enzymes on the improvement of the shear bond strength of restorative materials to dental structure [4, 5, 7–11]. Accordingly, the objective of the current study was to investigate the effects of 10% papain and 6% bromelain enzymes on the shear bond strength (SBS) of composite resin to enamel.

## 2. Material and Methods

### 2.1. Specimen Preparation

**Figure 1:**
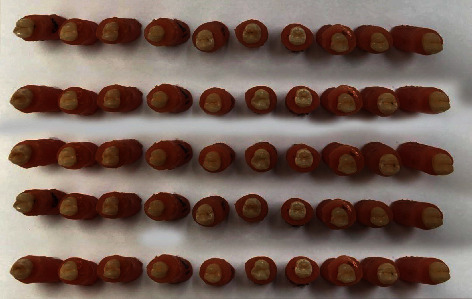
The prepared specimens of the study groups.

The study protocol was approved by the ethics committee of Shiraz University of Medical Sciences, Shiraz, Iran (IR.SUMS.REC.1396.S860). A total of 50 human maxillary premolar teeth without any cracks, restorations, caries, fractures, or stains extracted for orthodontic purposes were collected. Any remaining soft tissues were removed from the tooth surface and then stored in a 0.1% thymol solution (pH = 7) (Merck, Darmstadt, Germany) at 4°C for one month [2, 12]. Afterwards, the teeth were rinsed, gently dried, and embedded in acrylic resin (Acropars, Marlic Medical Co., Tehran, Iran) to ensure that an occlusal surface was mounted parallel to the acrylic resin and the cementoenamel junction (CEJ) was 2 mm higher than the acrylic resin surface. The middle part of the labial surface at the height of contour was polished using 600-grit silicon carbide paper under constant water spray to homogenize the surface. The prepared specimens are shown in Figure 1.

### 2.2. Enamel Surface Pretreatment

The teeth were randomly divided into five groups (*n* = 10) as follows: G1: the enamel surface was etched with 37% phosphoric acid (DenFil® Etchant-37, Vericom, Anyang, Korea) for 15 seconds, washed with distilled water for 20 seconds, and air-dried; next, 10% papain enzyme (Organika Co., Richmond, Canada) was applied on the surface for 60 seconds using a microbrush (Regular Micro Applicator, Premium Plus International Ltd., Hong Kong) and washed with distilled water for 20 seconds; G2: the enamel surface was etched with 37% phosphoric acid for 15 seconds, washed with distilled water for 20 seconds, and air-dried; after that, 6% bromelain enzyme (Biozyme, Oldendorf, Germany) was applied on the surface for 60 seconds using a microbrush and washed with distilled water for 20 seconds; G3: 6% bromelain enzyme was applied on the enamel surface for 60 seconds using a microbrush instrument and washed with distilled water for 20 seconds; G4: 10% papain enzyme was applied on the enamel surface for 60 seconds using a microbrush instrument and washed with distilled water for 20 seconds; G5: the enamel surface was etched with 37% phosphoric acid for 15 seconds and washed with distilled water for 20 seconds.

### 2.3. Shear Bond Strength Testing

Following preparations, all specimens were air-dried and subjected to a two-step etch-and-rinse adhesive system (Adper Single Bond 2; 3M ESPE, St. Paul, MN, USA) according to the manufacturer's instructions. A nanohybrid composite (Z350; 3 M ESPE, St. Paul, MN, USA) was placed in 2 mm thick Teflon molds with 3 mm diameter and light-cured for 40 seconds using an LED curing light (Demi™ Plus, Kerr Dental, Bioggio, Switzerland) with light intensity at 1200 mW/cm^2^ and a wavelength of 470 nm throughout the study.

**Figure 2:**
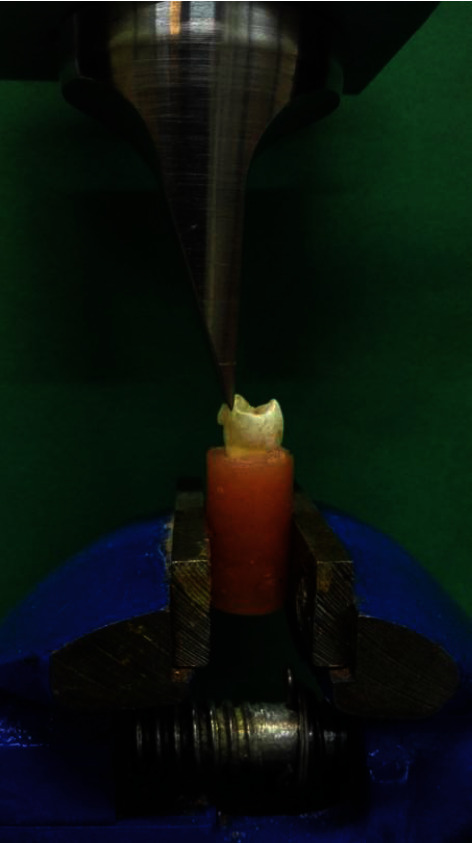
A prepared sample in the universal testing machine.

The specimens were stored in a 0.1% thymol solution for 24 hours at room temperature. Then, all the samples were subjected to the SBS test using a universal testing machine (Instron Z020, Zwick Roell, Ulm, Germany) at a crosshead speed of 1.0 mm/minute (Figure 2) [2]. The force was recorded in Newton and the SBS values were calculated in megapascal (MPa).

### 2.4. Statistical Analysis

Statistical analyses were performed using SPSS version 17 (SPSS Inc., Chicago, IL, USA). The normality of the data was checked using the Kolmogorov-Smirnov test. Afterwards, data analysis was performed using a one-way ANOVA. Post hoc comparisons of means were performed with the Tukey test. *p* values less than 0.05 were considered significant.

## 3. Results

**Figure 3:**
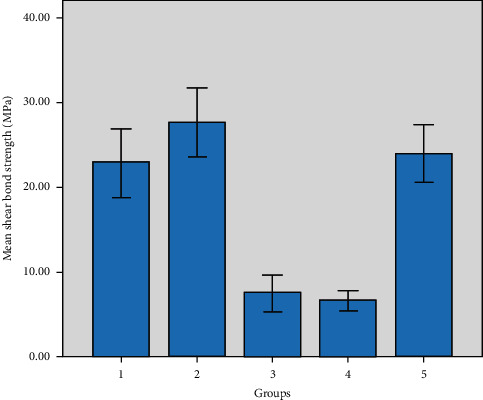
Means of the shear bond strengths (MPa) in the study groups. 1: (G1) 37% phosphoric acid and 10% papain. 2: (G2) 37% phosphoric acid and 6% bromelain. 3: (G3) 6% bromelain. 4: (G4) 10% papain. 5: (G5) 37% phosphoric acid.

The descriptive statistics of the SBSs of the study groups are presented in Table 1. The one-way ANOVA test was performed to compare the mean shear bond strength values among the study groups. The results indicated that the SBS was significantly influenced by the application of 37% phosphoric acid (*p* value < 0.05; Figure 3). Comparing the mean SBSs of G1, G2, and G5, no significant differences (*p* > 0.05) were observed. There was no significant difference between G3 and G4. The mean SBSs in Groups 1, 2, and 5 were significantly higher compared with groups where 6% bromelain or 10% papain was utilized without etching with 37% phosphoric acid. Besides, the group etched with 37% phosphoric acid and deproteinized with bromelain (G2) obtained the best result. The mean SBS values were further compared among the study groups (Table 2).

## 4. Discussion

According to the outcome of the present study, no significant difference was observed regarding the SBS between the group etched with 37% phosphoric acid and 10% papain enzyme (G1) and those only etched with 37% phosphoric acid (G5).

In line with our results, Hasija et al. compared the effect of papain gel on the SBS of composite resin to primary teeth enamel and found no statistically significant difference between the groups [11]. In contrast to the results of the present study, some previous studies indicated that enamel deproteinization with papain gel increased the shear bond strength, irrespective of the acid phosphoric etching application [5, 8, 10]. Eliminating the organic substances from the enamel surface before acid etching increases resistance by providing a better acid etching pattern on enamel. It seems that etching patterns before or after the application of papain have different results concerning SBS. Almost all the previous studies deproteinized enamel with papain enzyme followed by etching with 37% phosphoric acid, resulting in the highest SBS [5, 8, 10]; however, in the current study, the enamel surface was etched with 37% phosphoric acid and papain was further applied on the surface. This hypothesis is corroborated by a previous study in which higher SBS in the group deproteinized with 10% papain gel before acid etching was observed compared to SBS of the group deproteinized with 10% papain gel after acid phosphoric etching [8]. Etching enamel with 37% phosphoric acid after eliminating the organic elements from the enamel surface generates longer adhesive tags that penetrate the enamel. Also, the activity of phosphoric acid on the enamel surface occurs mostly on the mineralized tissues (inorganic matter). However, this acid does not eliminate organic materials [13–15].

The group etched with 37% phosphoric acid and deproteinized with bromelain enzyme (G2) obtained the best SBS in comparison with the other groups; however, the differences are not significant in comparison with G1 and G5. The mean SBS of the bromelain-treated group after application of 37% acid phosphoric was not significantly different from that of the group only etched with 37% phosphoric acid (G5), which is in agreement with a previous study [11]. The similarity of the results may be due to the use of bromelain after the acid etching process. On the other hand, Pithon et al. [4] suggested that enamel deproteinization with 6% bromelain in combination with 10% papain, when acid etching is performed with phosphoric acid, significantly increased the SBS, which is in contrast to our findings. It seems that the step of application of acid phosphoric and bromelain enzyme is the main factor to remove mineral content of enamel surface. Moreover, Chauhan et al. [9] reported that dentin deproteinization and removal of unsupported collagen fiber with bromelain enzyme after acid etching was able to statistically improve the SBS of the adhesive system. Sharafeddin et al. evaluated the effect of 10% papain and 6% bromelain enzymes on bond strength to superficial dentin using different adhesive systems [3]. They concluded that bond strength could be affected by the dissolution of collagen fibrils and change of the fibril composition may also be an effective factor in monomer diffusion by increasing dentin permeability [3]. Therefore, the type of the adhesive system and the deference in the composition of enamel and dentin could be an important factor on SBS when using bromelain and papain enzymes on tooth structure [3].

The concentration of papain (10%) or bromelain (6%) enzymes used in the current study is similar to those in the previous investigations that reported the effects of these agents on increasing the SBS [4, 5, 8, 10]. Furthermore, all specimens in the current study were prepared with one type of adhesive system and composite resin; accordingly, other materials may have different performances as observed in the previous studies using a combination of either papain (10%) or bromelain (6%) enzymes with Transbond XT bonding system and RMGIC [4, 5, 10]. Also, etching quality depends on the etching agent, acid concentration, etching time, and composition of the enamel surface [15]. Numerous studies have evaluated the effects of sodium hypochlorite (NaOCl) on the adhesion process. Sodium hypochlorite may exert different effects on bond strength depending on the chemical structure of the adhesive system and the type of the initiator in the adhesive system [16–18]. Some studies have revealed that the SBS of the enamel and dentin is enhanced by etching dental substrates with phosphoric acid [19–23], yet others have indicated that the pretreatment of enamel with phosphoric acid before the application of two-step self-etch adhesives may reduce the bond strength values [24, 25]. Ramakrishna et al. [6] showed that, after acid etching, enamel deproteinization had no improving effects on the SBS of the adhesive resin and composite resin to the treated enamel surface because preliminary acid-etching step with 37% phosphoric acid can exaggerate the enamel demineralization [21, 26]; therefore, the application of deproteinizing agents with lower acidity (such as papain or bromelain enzymes) after acid etching process with phosphoric acid could not significantly increase the SBS as observed in this study.

Because the applications of the deproteinizing agents without acid etching did not result in improved enamel bond strength compared to acid etching application in the present study, it is recommended to use acid etching for enamel bonding even when deproteinizing agents have been used. It has been reported that 3% bromelain enzyme can be as effective as 4% titanium tetrafluoride, 5% sodium hypochlorite, and 37% phosphoric acid on dentin bond strength [2]. Therefore, it is suggested to evaluate the effects of alternative pretreatments such as 4% titanium tetrafluoride and 5% sodium hypochlorite on enamel bond strength in future studies.

Mean bond strength values ranged between 7 and 27 MPa in the present report. Although no clear guidelines about shear force limits have been previously mentioned in the literature, a good orthodontic biomaterial should allow good adhesion to have enough resistance against sustaining masticatory forces (with a minimum bond strength of 5–10 MPa) [27]. On the other hand, adhesion forces should not be too strong to prevent substrate loss after debonding (40–50 MPa) [28]. Therefore, bonding forces should be in the interval of 5–50 MPa for an ideal orthodontic biomaterial, even if these limits are mostly theoretical [29].

Although this study is the first to evaluate the effects of bromelain and papain enzymes on the SBS of composite resin to enamel, the present study has some limitations. All specimens were tested with one type of adhesive system and composite resin, and using other materials or other test methods such as microshear and microtensile bond strength tests can have different performances. From a clinical point of view, some related factors in the oral environment can influence the results of the present laboratory report. In fact, the use of bioactive compounds and biomimetic remineralizing agents has a significant effect on the bond strength of restorative materials to the tooth structure and their mechanical properties [30, 31]. Therefore, future in vitro and clinical studies are needed to assess these unexplored variables. Furthermore, the effects of self-etch bonding were not evaluated on deproteinization with bromelain and papain enzymes; therefore, the effects of these deproteinizing agents on self-etch adhesive systems are questionable. Additionally, in the present research, the concentrations of bromelain and papain enzymes were selected based on a previous study [3]. More investigations with larger sample sizes and different concentrations of bromelain and papain enzymes using more precise tests such as microshear and microtensile bond strength tests are also required. It seems that future investigations should be focused on the elimination of the effects of acid etching or use acid etching following the application of bromelain or papain enzymes. Moreover, the effects of bromelain and papain enzymes on the shear bond strength of composite resin to enamel after the aging of the sample should be assessed in future studies.

## 5. Conclusion

The SBSs of all groups etched with 37% phosphoric acid are clinically acceptable; however, the SBS of composite resin to enamel was not affected significantly by etching with 37% phosphoric acid followed by the use of either 6% bromelain or 10% papain enzymes, compared to etching with 37% phosphoric acid alone. Moreover, 6% bromelain and 10% papain enzymes were not as effective as 37% phosphoric acid alone.

## Figures and Tables

**Table 1:** The mean shear bond strength (±SD) of the study groups.

Study groups	Shear bond strength (MPa)		
	Mean ± SD	Minimum	Maximum
Group 1 (37% phosphoric acid and 10% papain)	22.87 ± 4.02	18.20	29.20
Group 2 (37% phosphoric acid and 6% bromelain)	27.70 ± 4.14	21.60	31.70
Group 3 (6% bromelain)	7.44 ± 2.21	5.19	11.60
Group 4 (10% papain)	6.66 ± 1.19	5.07	8.81
Group 5 (37% phosphoric acid)	23.93 ± 3.38	19.00	27.60

**Table 2:** Comparison of mean shear bond strength values between the study groups.

Study groups	Group 1	Group 2	Group 3	Group 4	Group 5
Group 1 (37% phosphoric acid and 10% papain)	—	*p*=0.154	*p* < 0.0001	*p* < 0.0001	*p*=0.999
Group 2 (37% phosphoric acid and 6% bromelain)	*p*=0.154	—	*p* < 0.0001	*p* < 0.0001	*p*=0.332
Group 3 (6% bromelain)	*p* < 0.0001	*p* < 0.0001	—	*p*=0.985	*p* < 0.0001
Group 4 (10% papain)	*p* < 0.0001	*p* < 0.0001	*p*=0.985	—	*p* < 0.0001
Group 5 (37% phosphoric acid)	*p*=0.999	*p*=0.332	*p* < 0.0001	*p* < 0.0001	—

## Data Availability

The data that support the findings of this study are available upon request from the corresponding author.
